# *Pasteurella* Infections in South Korea and Systematic Review and Meta-analysis of *Pasteurella* Bacteremia 

**DOI:** 10.3201/eid3010.240245

**Published:** 2024-10

**Authors:** Seri Jeong, Eunjin Chang, Nuri Lee, Hyun Soo Kim, Han-Sung Kim, Jae-Seok Kim, Young Ah Kim, Chang Ki Kim, Kyungwon Lee, Hyukmin Lee, Seok Hoon Jeong, Wonkeun Song

**Affiliations:** Hallym University College of Medicine, Seoul, South Korea (S. Jeong, E. Chang. N. Lee, J.-S. Kim, W. Song);; Hallym University College of Medicine, Hwaseong, South Korea (H.S. Kim);; Hallym University College of Medicine, Anyang, South Korea (H.-S. Kim);; Ilsan Hospital, Goyang, South Korea (Y.A. Kim);; Seoul Clinical Laboratories Academy, Yongin, South Korea (C.K. Kim, K. Lee);; Yonsei University College of Medicine, Seoul (K. Lee, H. Lee, S.H. Jeong)

**Keywords:** Pasteurella, Pasteurella multocida, bacteremia, prevalence, meta-analysis, systematic review, zoonoses, South Korea, bacteria

## Abstract

*Pasteurella* spp. can cause fatal zoonotic infections in humans. We performed a multicenter study to investigate the prevalence and clinical features of *Pasteurella* infections in South Korea during 2018‒2022. We also conducted a collaborative systematic review and meta-analysis of the global burden of *Pasteurella* bacteremia. The study included 283 cases found an increasing trend in *Pasteurella* infections. Blood cultures were positive in 8/35 (22.9%) cases sampled, for overall bacteremia-associated rate of 2.8% (8/283). Aging was a significant risk factor for bacteremia (odds ratio 1.05 [95% CI 1.01–1.10]), according to multivariate analyses. For the meta-analysis, we included a total of 2,012 cases from 10 studies. The pooled prevalence of bacteremia was 12.4% (95% CI 7.3%–18.6%) and of mortality 8.4% (95% CI 2.7%–16.5%). Our findings reflect the need for greater understanding of the increase in *Pasteurella* infections and the global burden of *Pasteurella* bacteremia to determine appropriate case management.

*Pasteurella* spp. can cause fatal zoonotic infections in humans (*1*,*2*). *Pasteurella* spp., which are nonmotile, facultatively anaerobic bacteria, form the oral and gastrointestinal flora of many animals including companion and common livestock animals such as dogs, cats, and pigs (*3*–*5*). The health risk for humans and public health concerns regarding *Pasteurella* spp. should not be ignored when considering the increase in the numbers of those animals, caused by global economic and social development (*1*,*6*,*7*).

Strains of *P. multocida*, one of the most commonly isolated *Pasteurella* pathogens, have the capacity to invade human bronchial epithelial cells (*1*,*2*). The characteristics of human *Pasteurella* infections range from the commonly reported localized infection of a bite wound (*8*,*9*) to invasive infections such as bacteremia (*10*,*11*), meningitis (*12*), and infective endocarditis (*13*), especially in immunocompromised patients. Those invasive infections are associated with higher mortality rates in patients with pasteurellosis (*10*,*14*). However, only a few cohort studies on the epidemiology and clinical characteristics of *Pasteurella* infections have been published, mostly in Europe and the United States. Most studies regarding *Pasteurella* infections are reports of individual cases. Furthermore, the number of companion animals and the occurrence of humans having close contact with such animals have increased substantially (*15*).

We conducted a multicenter study of infections caused by *Pasteurella* spp. from various locations in South Korea during 2018‒2022 to investigate their prevalence and clinical features. In addition, we conducted a comprehensive systematic review and meta-analysis to characterize the global burden of bacteremia as a representative disease of invasive infection caused by *Pasteurella* spp. We performed subgroup analyses stratified by publication periods and study locations. The Institutional Review Board of Kangnam Sacred Heart Hospital, Seoul (HKS 2023-01-008) approved the study and waived the need for informed consent because of participant anonymity.

## Methods

### Ethics

### Study Design and Patients

**Figure 1:**
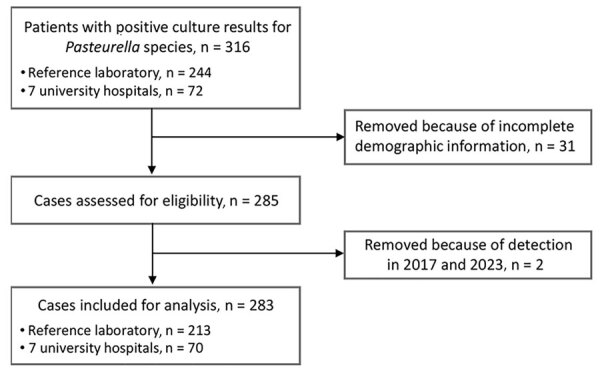
Flow diagram of study population selection for study of *Pasteurella* infection, South Korea, 2018–2022.

We designed a retrospective multicenter study of the prevalence of infections caused by *Pasteurella* spp. combined with a meta-analysis to determine the burden of these infections, especially bacteremia. We obtained data for *Pasteurella* species infections during 2018‒2022 from 7 university hospitals in metropolitan areas (4 in Seoul and 3 in Gyeonggi-do) and 1 reference laboratory, where samples from general and small- and medium-sized hospitals were tested, in South Korea to investigate the overall burden of these infections throughout the country. Patients were included if they had a microbiological examination that was positive for *Pasteurella* species. We identified isolated species by matrix-assisted laser desorption/ionization time-of-flight mass spectrometry on a Vitek-MS instrument (bioMérieux) or Bruker instrument (Bruker Daltonik), Vitek 2 system (bioMérieux), and the MicroScan Walkaway-96 system (Siemens). We obtained the following clinical variables from patient charts: basic demographics, hospital region of origin, sampling year, isolation site, the presence of polymicrobial infection, any animal contact such as bite or scratch history, and the antibiotics and therapies used. We also collected data for hospitalization and outcomes. From the 316 participants, we excluded 31 whose records lacked basic demographic information, such as age and sex (Figure 1). We included a total of 283 patients after excluding 2 patients whose *Pasteurella* species were isolated in 2017 and 2023. We obtained only basic data—age, sex, sampling year, isolation site, and region—for 213 participants from the reference laboratory.

### Search Strategy and Selection Criteria for Meta-analysis

We performed a systematic review as described in the Cochrane handbook (*16*) to estimate the global prevalence of bacteremia caused by *Pasteurella* spp. We referred to the Preferred Reporting Items for Systematic Review and Meta-analysis (*17*,*18*) checklist (Appendix Table 1). We included studies that reported prevalence data for patients with *Pasteurella* species infection and bacteremia based on laboratory results. We included observational cohort studies, regardless of language or publication year. We excluded studies without the necessary data for the calculation of the prevalence of bacteremia (Appendix Table 2).

We performed a comprehensive search of PubMed, Ovid-EMBASE, and the Cochrane Library for articles published through November 1, 2023. The search strategy included use of the keywords “Pasteurella infections,” “bacteremia,” “prevalence,” and “epidemiology.” We included Medical Subject Heading and Emtree terms, text words, and equivalent subject heading and thesaurus terms to ensure inclusivity; we also performed manual searches of the references of relevant articles for completeness (Appendix Table 3). We registered the protocol in the international prospective register for systematic reviews (registration no. CRD42023484039).

### Analysis of the Study Population Data and Meta-analysis

For statistical analysis of data from multiple centers in South Korea, we used the Mann-Whitney U test or Pearson’s χ^2^ test to compare groups. We applied multivariate binary logistic regression analyses to investigate variables that correlated independently with the occurrence of bacteremia in patients with *Pasteurella* spp. infections.

**Figure 2:**
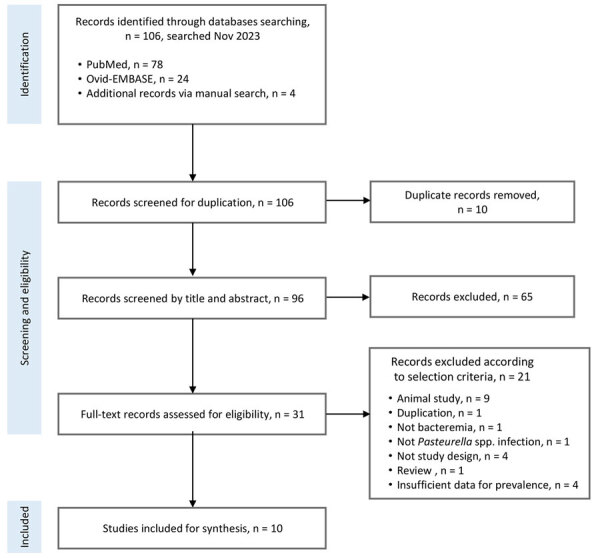
Flow diagram of study selection process in systematic review and meta-analysis of studies of human bacteremia caused by *Pasteurella* spp..

For the meta-analysis, we conducted title and abstract screening of studies selected through the search strategy on the basis of the eligibility criteria (Figure 2). Two reviewers (E.J. and N.L.) independently assessed the full texts of the studies. We settled disagreements by consensus after all reviewers reviewed the data. We extracted the following variables if they were available: demographic information about the study population, collection periods, the presence of animal exposure, the identified species, regions, and outcome measures. We documented he data for hospitalization and death, as well as the prevalence of bacteremia, our primary outcomes. We used the Joanna Briggs Institute checklist (*19*) to evaluate the quality of the included articles at the study level. We considered scores >70% as high quality. Two reviewers (E.J. and N.L.) assessed the quality of the included studies. S.J. resolved any disagreements.

We calculated the proportion of patients with positive blood cultures for the determining the rate of bacteremia among the total number of patients with bacterial infections. We based the calculation on all types of samples for which culture tests were requested (*3*,*4*,*10*,*20*). Although simultaneous blood culture is necessary for a more accurate determination of the rates of bacteremia, it was not routinely performed. Blood culture was performed only in 35/283 cases in which the clinician deemed it necessary. We transformed single raw prevalence using the Freeman-Tukey Double arcsine method (*21*) to stabilize variances. We used the random effects model to calculate the pooled prevalence with 95% CI across studies.

We assessed heterogeneity using Cochran’s Q test and the degree of heterogeneity using the Higgins *I*^2^ statistic. Indices with values of >75% represented high heterogeneity (*22*). We performed subgroup analyses to explore potential sources of heterogeneity by study location and period. In addition, we conducted sensitivity analysis to further assess the robustness of the estimates. The software we used for those analyses was the moonBook package in R (The R Project for Statistical Computing), MedCalc software, version 19.8 (MedCalc Software Ltd), Analyze-it Method Evaluation Edition software version 2.26 (Analyze-it Software Ltd), and Stata version 18 (StataCorp LLC). We have deposited the raw data used in this study (Appendix Tables 4, 5) in the Harvard Dataverse (https://doi.org/10.7910/DVN/1QQ9KK).

## Results

### Prevalence and Clinical Features of *Pasteurella* Infections

**Figure 3:**
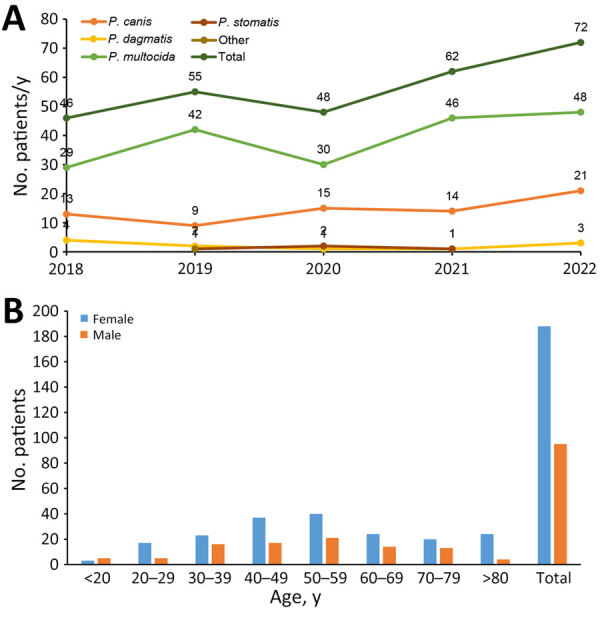
Prevalence of *Pasteurella* infections in South Korea, 2018–2022. A) Distribution of *Pasteurella* infections classified by year and species. B) Distribution of *Pasteurella* infections classified by age group and sex.

We included a total of 283 cases in the study, 70 from hospital patients with complete data and 213 cases from the main reference laboratory with basic information. We observed an increase in the number of infections caused by *Pasteurella* spp. from 2018 (n = 46) to 2022 (n = 72) (Figure 3); the increase was significant on the basis of the national population data extracted from the Korean Statistical Information Service (p = 0.012). The median number of cases per year was 55. The predominant species were *P. multocida* (68.9%, 195/283) and *P. canis* (25.4%, 72/283), both of which contributed to the increase in infections. The median age of patients with positive culture results was 52.0 years. The number of *Pasteurella* isolation samples was higher in women (66.4%, 188/283) than in men (33.6%, 95/283). We observed the most cases in patients in the 50‒59-year age group (Figure 3). The ratio of female to male patients was the highest (3.4:1) for patients 20‒29 years of age. Most patients had a history of companion animal exposure (88.6%, 62/70) (Appendix Table 6); half had history of dog exposure and 38.7% (24/62) cat exposure. The rate of polymicrobial infection was 25.7% (18/70). The detected isolates were *Staphylococcus aureus* (n = 2), *Actinomyces* spp. (n = 2), *Streptococcus sanguinis* (n = 1), *Proteus mirabilis* (n = 1), and *Dermabacter hominis* (n = 1). The rate of hospitalization was 54.3% (38/70). We noted no significant differences in characteristics between inpatients and outpatients (Appendix Table 7). Among all the patients, most patients were mainly from Seoul (23.0%, 65/283), whereas those included in the reference laboratory study were predominantly from Gyeongsang-do (33.0%, 71/213) and Gyeonggi-do (17.4%, 37/213) (Appendix Figure 1). All patients, except for 3 outpatients, had received antimicrobial therapy. The most frequently prescribed antibiotics were penicillin/β-lactamase inhibitor combinations and first-generation cephalosporins (Appendix Table 4).

**Figure 4:**
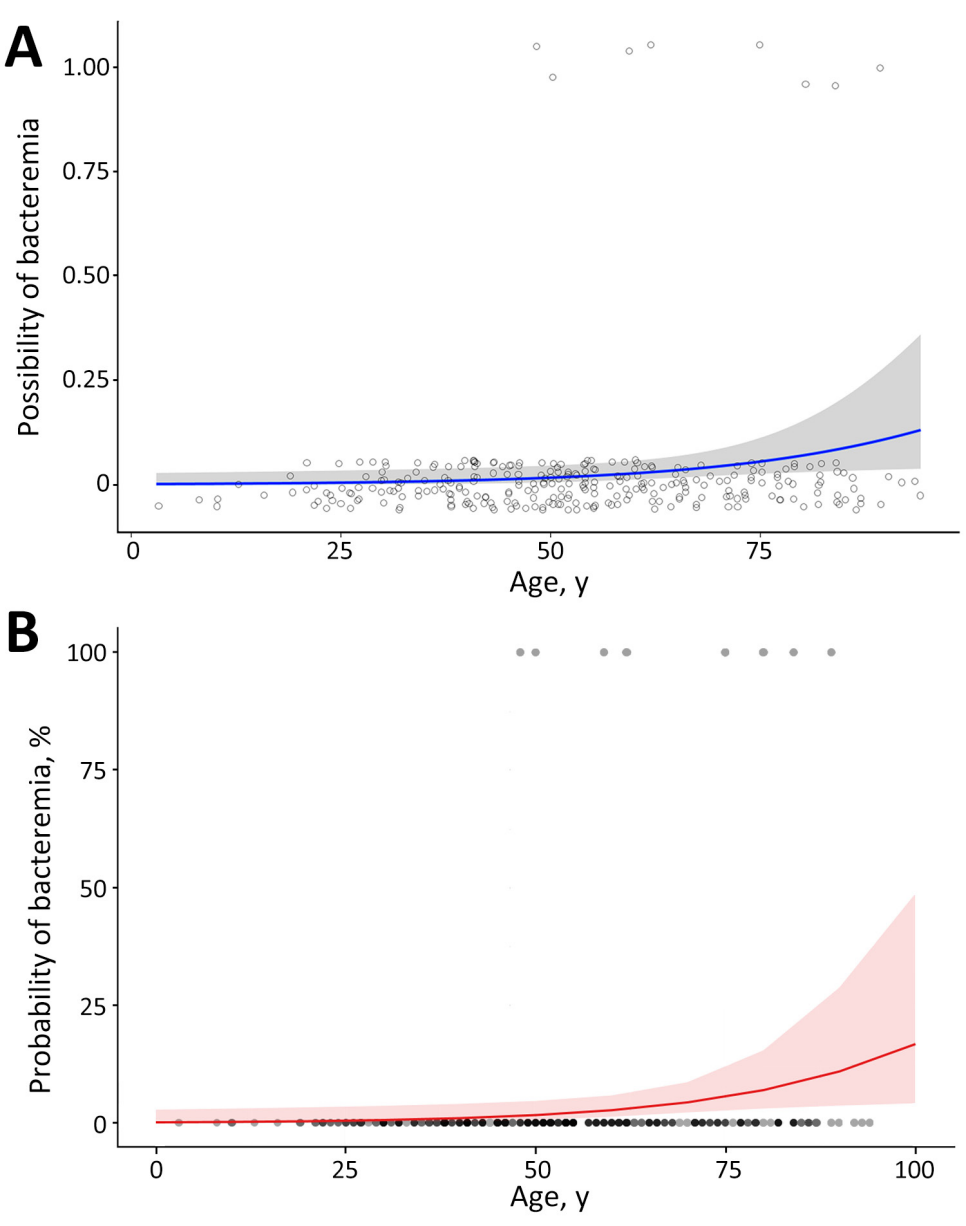
Regression model of bacteremia caused by *Pasteurella* spp., South Korea, 2018–2022. A) Univariate analysis of age and the probability of bacteremia caused by *Pasteurella* spp. The smoothing method was a generalized linear model. Blue line indicates the estimated values of the possibility of bacteremia; shading indicates 95% CI; black circles indicate cases with bacteremia; and gray circles indicate cases without bacteremia. B) Final model after multivariate analysis of age and sex for the predicted probability of bacteremia caused by *Pasteurella* spp. Red line indicates the estimated values of the probability of bacteremia; shading indicates 95% CI; black dots indicate cases with bacteremia; and gray dots indicate cases with non-bacteremia.

The rates of bacteremia were 2.8% (8/283) among all included infections and 7.1% (5/70) among the 7 university-hospital cases (Appendix Table 8). The median age was higher in bacteremia (68.5 years) than that in nonbacteremia (52.0 years). Patients with bacteremia had no animal exposure history. According to the multivariate regression analyses, including sex as the confounder, only increasing age was a significant risk factor for bacteremia (odds ratio 1.05, 95% CI 1.01–1.10; p = 0.024) (Figure 4). Among all bacteremia cases, 1 patient with septic shock caused by *P. multocida* died after 4 days of hospitalization; that patient had alcoholic liver cirrhosis and asthma as underlying diseases.

### Meta-analyses for Global Burden of *Pasteurella* Bacteremia

The study screening method for the meta-analysis (Figure 2) identified 106 studies, of which 96 articles were relevant and were subsequently screened. A total of 31 reports were available for full-text screening. We excluded papers with insufficient study populations and design and insufficient data for the calculation of the prevalence of *Pasteurella* spp. bacteremia from the current review (Appendix Table 2). Finally, we included 10 studies in the systematic review (*3*,*4*,*6*,*7*,*10*,*20*,*23*–*26*).

We included a total of 2,012 participants from the 10 studies in the meta-analysis (Table). The studies were published during 1985‒2021, half before 2010 (*4*,*10*,*20*,*25*,*26*) and half after 2010 (*3*,*6*,*7*,*23*,*24*). We observed more than 50% of study participants were female in all the included studies. The occurrence of animal exposure calculated from available study data was 34.3% ‒97.3%. The most commonly isolated species were *P. multocida* and *P. canis*. The rates of hospitalization were higher in cases of invasive infection (83.3% [*23*] and 97.0% [*24*]) than those in other cases (*3*,*6*,*7*,*20*). The death rates range was 0.6%‒27.2%. Six studies were conducted in Europe and the United States (*3*,*7*,*10*,*20*,*23*–*26*). Based on the assessment using the Joanna Briggs Institute checklist, all studies showed scores >70%, indicating that they were of high quality. Four studies that included only *P. multocida* infections had lower scores in the samples frame appropriate to address the target population (*3*,*4*,*20*,*25*).

**Table:** Characteristics of study of *Pasteurella* infections in South Korea and 10 studies included in meta-analysis of bacteremia caused by *Pasteurella* spp.*

Study location	No. patients	Collection period	Age, y†	Sex ratio, M:F	No. (%) with animal exposure	Species (no.)	Hospitalizations, no. (%)	No. (%) deaths	Ref
South Korea	283‡	2018–2022	52.0	95:188	62/70‡ (88.6)	*P. multocida* (195), *P. canis* (72), *P. dagmatis* (11), *P. stomatis* (4)	38/70‡ (54.3)	1/70‡ (1.4)	This study
Greece	13	1993–2004	64.4	10:3	5 (38.5); 2 unknown	*P. multocida* (13)	NR	3 (23.1)	(*25*)
France	215: 45 invasive, 170 local	2005–2018	59.8 for invasive, 49.1 for local	29:16 for invasive, 64:106 for local	16 of invasive, 21 of complicated local	*P. multocida* (169/215 total), *P. canis* (32/170 local)	65/67 (97.0) invasive and complicated local	10/45 (22.2) invasive	(*23*)
United States	179	1987–2007	66	6:8	7 (50.0) of 14 hospitalized	*P. multicida* (179)	14 (7.8)	1 (0.6)	(*20*)
France	958	1985–1991	NR	NR	35/102§ (34.3)	*P. multocida* (460), *P. canis* (105), *P. dagmatis* (48), *P. stomatis* (38)	NR	12/87 (13.8) with septicemia	(*10*)
United States	44	2000–2014	64	14:30	25 (56.8)	*P. multocida* (44)	27 (61.4)	4 (9.1)	(*3*)
Denmark	146	1989–1992	NR	NR	142 (97.3)	*P. multocida* (95), *P. canis* (28), *P. septica* (21), *P. stomatis* (10), *P. dagmatis* (5)	NR	NR	(*26*)
Hungary	162	2002–2015	57	78:84	114 (70.4)	*P. multocida* (160), *P. canis* (36), *P. pneumotropica* (11)	71/114 (62.3) local, 40/48 (83.3) invasive	44 (27.2)	(*24*)
Australia	190	2000–2021	49.7	93:97	145 (76.3)	*P. multocida* (121), *P. canis* (45), *P. dagmatis* (2)	148 (77.9)	2 (1.1)	(*6*)
France	102: 74 local, 28 invasive	2000–2015	63 for invasive, 50 for local	38:64	NR	*P. multocida* (86), *P. canis* (10), *P. dagmatis* (1), *P. stomatis* (1)	75 (73.5)	4 (3.9)	(*7*)
Israel	77	2000–2005	49.2	38:39	46 (59.7)	*P. multocida* (77)	NR	2 (2.6)	(*4*)

*NR, not reported; ref, reference. †Mean or median, as reported in article. §15 cases of bacteremia and 87 cases of septicemia. ‡70 hospital patients with complete data and 213 cases from the main reference laboratory records.

**Figure 5:**
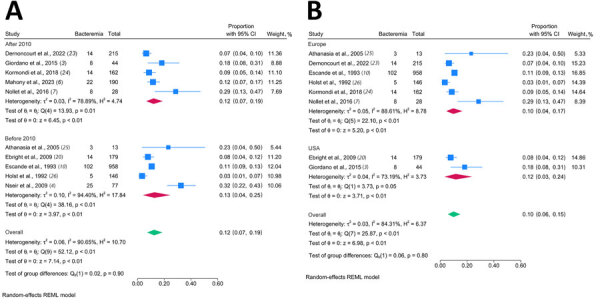
Forest plots for the pooled prevalence rates in systematic review and meta-analysis of studies of human bacteremia caused by *Pasteurella* spp. A) Subgroup analysis by year study was published. B) Subgroup analysis by study location. Blue squares indicate the rates of bacteremia of the included studies; error bars indicate 95% Cis. Red diamonds indicate the pooled rates of the included studies in the subgroup analysis, and green diamonds indicate the pooled rates of all studies presented in each subgroup analysis.

The prevalence of bacteremia caused by *Pasteurella* spp. from 10 studies was 3.4%‒32.5%. The random effects pooled prevalence from the 2,012 cases was 12.4% (95% CI 7.3%–18.6%). The Cochran Q test revealed significant heterogeneity (Q = 52.1, p<0.001). The *I*^2^ index of the included studies indicated high heterogeneity (*I*^2^ = 90.7) (Figure 5).

We calculated the pooled prevalence estimates of *Pasteurella* infection stratified by publication year. The pooled value of 5 studies published before 2010 (*4*,*10*,*20*,*25*,*26*) was 12.7% (95% CI 4.1%–24.6%) and of 5 studies published after 2010 (*3*,*6*,*7*,*23*,*24*) was 12.1% (95% CI 6.6%–18.9%). Although the *I*^2^ index for studies published after 2010 (78.9%) was lower than that for studies before 2010 (94.4%), we noted no significant differences in pooled prevalence and heterogeneity. In a subgroup analysis of different regions, the estimated prevalence infection in 6 studies conducted in Europe (9.8%, 95% CI 4.5%–16.6%) was not different from that of 2 studies conducted in the United States (11.6%, 95% CI 3.3%–23.6%). We excluded 1 of the 10 studies from the sensitivity analyses. We compared estimated prevalence to the estimated total prevalence (Appendix Table 9). The pooled estimates from the sensitivity analyses were 9.9%‒13.8%, which was consistent with the total pooled prevalence without statistical differences. The pooled death rate was 8.4% (95% CI 2.7%–16.5%) (Appendix Figure 2).

## Discussion

In this analysis of data from 8 centers in South Korea and a meta-analysis of the global burden of *Pasteurella* infections, we included 283 cases of infection caused by *Pasteurella* spp. in South Korea and 2,012 cases from 10 previous studies. We observed an increasing trend of *Pasteurella* infections during 2018‒2022; South Korea had a median of 55 cases/year. The increasing trend of *Pasteurella* infections we observed in this study was consistent with the results of studies conducted in Australia (*6*) and Hungary (*24*). Those findings can be attributed to the increasing number of companion animals and their close contact with humans in South Korea. In Canada, ≈57% of households have >1 companion animal (*27*). The mean number of animal bites per year in Israel is 15,000, according to data from the Israeli Ministry of Health (*4*). In the United States, emergency departments observe ≈3 million dog bite injuries that lead to 10,000 hospitalizations and 20 deaths annually (*28*).

Among *Pasteurella* species, *P. multocida* is the most frequently isolated, followed by *P. canis* (*6*,*7*,*10*,*23*,*24*). Our results were consistent with those findings. The polymicrobial nature of *Pasteurella* infection was persistently reported. Mahony et al. (*6*) reported that 23.8% of cases exhibited polymicrobial infection, which was similar to the rate (25.7%) we observed in our study *Staphylococcus aureus*, *Actinomyces* spp., *Streptococcus sanguinis*, and *Dermatobia hominis*, which were the co-isolated strains in this study, usually act as commensal bacteria in humans. *S. aureus* has been frequently isolated in several studies (*6*,*24*), concordant to our results.

For the demographic distribution, our study confirmed the predominance of infections in female patients, which has been consistently observed in previous studies, despite some variations (*3*,*7*,*24*). Dernoncourt et al. (*23*) observed this female predominance in localized infection cases rather than invasive infection cases, which might be associated with the higher proportions of localized infection we observed. Patients 50‒59 years of age exhibited the most infections in this study. A survey in Canada found that the rate of pet ownership was highest among middle-aged persons (*27*), which supports our results, considering that pet-associated infections are frequently derived from injuries or animal bites (*24*). The high ratio of female to male patients 20‒29 years of age could be because the primary responsibility for the care of companion animals is mostly that of female persons (72.8%) within households (*29*); another possible cause is that some nonmarried women spend substantial time with their cats (*29*).

Regarding bacteremia, older age groups are associated with bacteremia caused by *Pasteurella* spp. (*4*,*6*,*7*), consistent with the results of our study. Age-related dysfunction of the immune system and underlying diseases contribute to the increased risk for invasive infections (*24*). Underlying conditions such as diabetes mellitus and cirrhosis were commonly reported risk factors for bacteremia (*11*,*30*). Nollet et al. (*7*) determined by univariate analysis that chronic liver disease and alcohol consumption were risk factors for invasive *Pasteurella* infection; however, multivariate analysis showed that age was a significant risk factor (*7*). For animal contact, bacteremia was associated with the absence of animal bites or contacts (*7*,*24*), similar to our results. A previous study reported acute epiglottis without animal exposure (*31*), and a review described 79 cases including 34 of nonbite transmission (*14*). This type of transmission was related to comorbidities resulting in life-threatening infections. For example, contaminating a metatarsal ulcer by stepping on dog drool or wearing socks covered with cat hair could lead to bacteremia. The protection of open wounds is necessary for prevention because they were the most common entry method for non–bite-associated infections.

Several case reports or reviews have described severe systemic infections caused by *Pasteurella* spp., such as bacteremia and endocarditis. A review of *P. multocida* bacteremia presented the clinical features and outcome of 13 patients (*32*). A recent review focused on epidemiology, diagnosis, host‒pathogen interactions, clinical manifestation, management, and prognosis of *P. multocida* infections (*2*). In addition, a systematic review of infective endocarditis caused by *Pasteurella* species described the clinical characteristics and outcomes of patients on the basis of data from 28 studies (*13*). However, meta-analyses with specified values have not been performed. Therefore, we conducted meta-analyses assessing the global burden of *Pasteurella* bacteremia as a representative invasive infection.

The pooled prevalence from 10 studies was 12.4%, which was higher than the rate of bacteremia observed in this study (2.8% from all episodes and 7.1% from 7 hospital cases). The study populations of the included studies included patients in tertiary and university hospitals (*3*,*6*,*23*–*26*) or with hospitalization (*4*), which may have influenced this high prevalence. In addition, high medical accessibility with a reimbursement system and health screenings for the elderly could be the cause of the significantly low prevalence of bacteremia we observed in our study. The high heterogeneity may be derived from the different periods of isolation and publication and by the variation across geographic regions of the country. We performed subgroup analyses stratified by publication year and study locations, which showed no significant differences, except for a slightly lower *I*^2^ index (from 94.4% before 2010 to 78.9% after 2010). The overlap of data collection periods caused by long (median 13 years) study durations might have affected these results. In addition, the small number of studies on *Pasteurella* bacteremia used for this analysis might have affected the statistical results.

The number of deaths caused by *Pasteurella* infection is increasing in the United States (*33*). Meta-analyses revealed that the pooled estimate of deaths was 8.4%, which was much higher than the death rate measured in our study (0.4% for all and 1.4% for hospital cases). The low rate in our study is consistent with the low prevalence of bacteremia in our cohort; the mortality rate for patients with invasive infections was higher (*10*,*23*). Although the rate of infections is low, mortality rate may increase as the prevalence of *Pasteurella* infection increases.

One limitation of this study was its retrospective nature; some clinical information documented in the medical record was incomplete or missing. In particular, only basic demographic information was available for patients from the reference laboratory. Therefore, we included data from hospitals; data from multiple centers are a strength of this study. However, diverse identification methods and the inherent limitations of the applied methods may affect the results (*12*). The relatively small size of our cohort might influence the statistical analysis. Studies with larger study populations, including hospitals in rural and farming areas, are necessary. Detailed descriptions of the methods used for calculating the bacteremia rate would be useful to estimate the specified rate of bacteremia; in addition, further studies with concurrent blood culture data are needed to determine more accurate rates of bacteremia. For meta-analyses, additional studies from diverse countries are necessary for generalization. We could not sufficiently analyze publication bias because we included a limited number of studies. In addition, the heterogeneity of the included studies may have affected the results; we conducted subgroup analyses, a random effects model, and sensitivity analyses to overcome this limitation. 

In conclusion, this study highlights the increasing trend and clinical features of *Pasteurella* infections, the rate of bacteremia, and older age as a risk factor for bacteremia based on data from 8 centers in South Korea. We estimated the global prevalence of bacteremia and related death rates through a collaborative approach with systematic meta-analysis. Our findings indicate that more attention needs to be paid to *Pasteurella* infection to enable appropriate management of these cases.

Appendix Additional information about *Pasteurella* infections in South Korea and systematic review and meta-analysis of *Pasteurella* bacteremia.
