# Supplementary material for: Pasteurella Infections in South Korea and Systematic Review and Meta-analysis of Pasteurella Bacteremia
Source: Emerg Infect Dis. 2024 Oct;30(10):1987–97. doi: 10.3201/eid3010.240245 (PMC11431893; doi:10.3201/eid3010.240245)
Supplement: Appendix — Additional information about Pasteurella infections in South Korea and systematic review and meta-analysis of Pasteurella bacteremia. [file 24-0245-Techapp-s1.pdf]

Article DOI: https://doi.org/10.3201/eid3010.240245

EID cannot ensure accessibility for supplementary materials supplied by authors. Readers who have difficulty accessing supplementary content should contact the authors for assistance.

Pasteurella Infections in South Korea and Systematic Review and Meta-analysis of *Pasteurella* Bacteremia

Appendix

Appendix Table 1. Preferred reporting Items for the systematic review and meta-analysis checklist

Section And Topic	Item #	Checklist item	Location where item is reported
Title			
Title	1	Identify the report as a systematic review.	Title of paper
Abstract			
Abstract	2	See the PRISMA 2020 for Abstracts checklist.	We have checked.
Introduction			
Rationale	3	Describe the rationale for the review in the context of existing knowledge.	Introduction, Paragraph 1 and 2
Objectives	4	Provide an explicit statement of the objective(s) or question(s) the review addresses.	Introduction, Paragraph 3
Methods			
Eligibility criteria	5	Specify the inclusion and exclusion criteria for the review and how studies were grouped for the syntheses.	Methods, Subsection “Search strategy and selection criteria for meta-analysis”
Information sources	6	Specify all databases, registers, websites, organisations, reference lists and other sources searched or consulted to identify studies. Specify the date when each source was last searched or consulted.	Methods, Subsection “Search strategy and selection criteria for meta-analysis”
Search strategy	7	Present the full search strategies for all databases, registers and websites, including any filters and limits used.	Table S3
Selection process	8	Specify the methods used to decide whether a study met the inclusion criteria of the review, including how many reviewers screened each record and each report retrieved, whether they worked independently, and if applicable, details of automation tools used in the process.	Methods, Subsection “Data analysis for study population and meta-analysis”
Data collection process	9	Specify the methods used to collect data from reports, including how many reviewers collected data from each report, whether they worked independently, any processes for obtaining or confirming data from study investigators, and if applicable, details of automation tools used in the process.	Methods, Subsection “Data analysis for study population and meta-analysis”
Data items	10a	List and define all outcomes for which data were sought. Specify whether all results that were compatible with each outcome domain in each study were sought (e.g. for all measures, time points, analyses), and if not, the methods used to decide which results to collect.	Methods, Subsection “Data analysis for study population and meta-analysis”
	10b	List and define all other variables for which data were sought (e.g. participant and intervention characteristics, funding sources). Describe any assumptions made about any missing or unclear information.	Methods, Subsection “Data analysis for study population and meta-analysis”

Section And Topic	Item #	Checklist item	Location where item is reported
Study risk of bias assessment	11	Specify the methods used to assess risk of bias in the included studies, including details of the tool(s) used, how many reviewers assessed each study and whether they worked independently, and if applicable, details of automation tools used in the process.	Methods, Subsection "Data analysis for study population and meta-analysis"
Effect measures	12	Specify for each outcome the effect measure(s) (e.g. risk ratio, mean difference) used in the synthesis or presentation of results.	Methods, Subsection "Data analysis for study population and meta-analysis"
Synthesis methods	13a	Describe the processes used to decide which studies were eligible for each synthesis (e.g. tabulating the study intervention characteristics and comparing against the planned groups for each synthesis (item #5)).	Methods, Subsection "Data analysis for study population and meta-analysis"
	13b	Describe any methods required to prepare the data for presentation or synthesis, such as handling of missing summary statistics, or data conversions.	Methods, Subsection "Data analysis for study population and meta-analysis"
	13c	Describe any methods used to tabulate or visually display results of individual studies and syntheses.	Methods, Subsection "Data analysis for study population and meta-analysis"
	13d	Describe any methods used to synthesize results and provide a rationale for the choice(s). If meta-analysis was performed, describe the model(s), method(s) to identify the presence and extent of statistical heterogeneity, and software package(s) used.	Methods, Subsection "Data analysis for study population and meta-analysis"
	13e	Describe any methods used to explore possible causes of heterogeneity among study results (e.g. subgroup analysis, meta-regression).	Methods, Subsection "Data analysis for study population and meta-analysis"
	13f	Describe any sensitivity analyses conducted to assess robustness of the synthesized results.	Methods, Subsection "Data analysis for study population and meta-analysis"
Reporting bias assessment	14	Describe any methods used to assess risk of bias due to missing results in a synthesis (arising from reporting biases).	Methods, Subsection "Data analysis for study population and meta-analysis"
Certainty assessment	15	Describe any methods used to assess certainty (or confidence) in the body of evidence for an outcome.	Methods, Subsection "Data analysis for study population and meta-analysis"
**RESULTS**			
Study selection	16a	Describe the results of the search and selection process, from the number of records identified in the search to the number of studies included in the review, ideally using a flow diagram.	Figure 1
	16b	Cite studies that might appear to meet the inclusion criteria, but which were excluded, and explain why they were excluded.	Results, Paragraph 3 and Figure 1
Study characteristics	17	Cite each included study and present its characteristics.	Results, Paragraph 4 and Table 2
Risk of bias in studies	18	Present assessments of risk of bias for each included study.	Results, Paragraph 4
Results of individual studies	19	For all outcomes, present, for each study: (a) summary statistics for each group (where appropriate) and (b) an effect estimate and its precision (e.g. confidence/credible interval), ideally using structured tables or plots.	Results, Paragraph 5 and Figure 4
Results of syntheses	20a	For each synthesis, briefly summarise the characteristics and risk of bias among contributing studies.	Results, Paragraph 4
	20b	Present results of all statistical syntheses conducted. If meta-analysis was done, present for each the summary estimate and its precision (e.g. confidence/credible interval) and measures of statistical heterogeneity. If comparing groups, describe the direction of the effect.	Results, Paragraph 5 and Figure 4
	20c	Present results of all investigations of possible causes of heterogeneity among study results.	Results, Paragraph 6 and Figure 4
	20d	Present results of all sensitivity analyses conducted to assess the robustness of the synthesized results.	Results, Paragraph 6 and Table S8
Reporting biases	21	Present assessments of risk of bias due to missing results (arising from reporting biases) for each synthesis assessed.	Results, Paragraph 6 and Table S8
Certainty of evidence	22	Present assessments of certainty (or confidence) in the body of evidence for each outcome assessed.	Results, Paragraph 6 and Figure 4
**DISCUSSION**			
Discussion	23a	Provide a general interpretation of the results in the context of other evidence.	Discussion, Paragraphs 1-8
	23b	Discuss any limitations of the evidence included in the review.	Discussion, Paragraphs 9

Section And Topic	Item #	Checklist item	Location where item is reported
	23c	Discuss any limitations of the review processes used.	Discussion, Paragraphs 9
	23d	Discuss implications of the results for practice, policy, and future research.	Discussion, Paragraphs 10
**OTHER INFORMATION**			
Registration and protocol	24a	Provide registration information for the review, including register name and registration number, or state that the review was not registered.	Abstract and Methods
	24b	Indicate where the review protocol can be accessed, or state that a protocol was not prepared.	Abstract and Methods
	24c	Describe and explain any amendments to information provided at registration or in the protocol.	Not applicable
Support	25	Describe sources of financial or non-financial support for the review, and the role of the funders or sponsors in the review.	Methods, Subsection “Role of the funding source” and Acknowledgments
Competing interests	26	Declare any competing interests of review authors.	Section “Declaration of interests”
Availability of data, code and other materials	27	Report which of the following are publicly available and where they can be found: template data collection forms; data extracted from included studies; data used for all analyses; analytic code; any other materials used in the review.	HARVARD Dataverse (https://doi.org/10.7910/DVN/1QQ9KK)

From: Page MJ, McKenzie JE, Bossuyt PM, Boutron I, Hoffmann TC, Mulrow CD, et al. The PRISMA 2020 statement: an updated guideline for reporting systematic reviews. BMJ 2021;372:n71. doi: 10.1136/bmj.n71

For more information, visit: http://www.prisma-statement.org/

Appendix Table 2. Excluded studies and the corresponding reasons

Reference (date)	Reason for exclusion	Reference
Chandranaik (2015)	Animal study	(*1*)
Chomnawang (2009)	Animal study	(*2*)
Dunbar (2000)	Animal study	(*3*)
Kawasaki (2015)	Animal study	(*4*)
Martrenchar (1994)	Animal study	(*5*)
Moustafa (2013)	Animal study	(*6*)
Qudratullah (2017)	Animal study	(*7*)
Sarangi (2016)	Animal study	(*8*)
Voigts (1997)	Animal study	(*9*)
Prakash (2009)	Duplication	(*10*)
Levy (1989)	Not bacteremia	(*11*)
Bardhan (2020)	Not *Pasteurella* species infection	(*12*)
Biswas (2004)	Not study design	(*13*)
MacPhillamy (2020)	Not study design	(*14*)
Mondal (2014)	Not study design	(*15*)
Tomer (2002)	Not study design	(*16*)
Kannangara (2020)	Review	(*17*)
Bhonsle (1951)	Insufficient data for prevalence	(*18*)
Carter (1982)	Insufficient data for prevalence	(*19*)
Martrenchar (1993)	Insufficient data for prevalence	(*20*)
Rimler (1994)	Insufficient data for prevalence	(*21*)

Appendix Table 3. Search strategy
(PubMed Search; adapted for other searches)
PubMed (2023.11.01)

No.	Query	Items found
#1	"Pasteurella Infections"[Mesh] OR "Pasteurella Infections"[TW] OR "Pasteurellosis"[TW] OR "Pasteurelloses"[TW] OR "Infections, Pasteurella"[TW] OR "Infection, Pasteurella"[TW] OR "Pasteurella Infection"[TW]	4,949
#2	"Bacteremia"[Mesh] OR "Bacteremia"[TW] OR "Bacteremias"[TW] OR "Septicemia"[TW] OR "Hemorrhagic Septicemia"[Mesh] OR "Hemorrhagic Septicemia"[TW]	63,552
#3	"Prevalence"[MeSH] OR "Epidemiology"[MeSH] OR "prevalence"[TW] OR "epidemiology"[TW] OR "incidence"[TW]	3,245,301
#4	#1 AND #2 AND #3	88
#5	#4 NOT ("Review"[Publication Type] OR "Review literature as topic"[MeSH])	78

Appendix Table 4. Dataset of infections caused by *Pasteurella* species from a multicenter study

Hospital	Identification	Year	Specimen	Site	Polymicrobial infection	Animal contact	Sex	Age	Region	Hospitalization	Used antibiotics	Outcome
Kangnam	P1	2018	Wound	Face	No	Yes	F	35	Gyeonggi-do	No	1. Ampicillin sodium/sulbactam sodium<br>2. Sultamicillin	Clinical cure
Kangnam	P2	2019	Wound	Upper extremity	No	Yes	F	51	Seoul	Yes	1. Ampicillin sodium/sulbactam sodium<br>2. Amox/clavulanic acid	Clinical cure
Kangnam	P3	2020	Wound	Upper extremity	No	Yes	M	80	Seoul	Yes	1. Ampicillin sodium/sulbactam sodium<br>2. Amox/clavulanic acid	Clinical cure
Kangnam	P4	2020	Wound	Other	No	Yes	F	26	Seoul	No	1. Ampicillin sodium/sulbactam sodium<br>2. Amox/clavulanic acid	Clinical cure
Kangnam	P5	2020	Blood	Blood	No	No	F	75	Gangwon-do	Yes	1. Ceftazidime<br>2. Ceftriaxone sodium<br>3. Vancomycin<br>4. Levofloxacin	Clinical cure
Kangnam	P6	2020	Wound	Face	Yes	Yes	F	32	Seoul	No	1. Cefalexin<br>2. Cefuroxime<br>3. Netilmicin	Clinical cure
Kangnam	P7	2020	Wound	Face	Yes	Yes	M	3	Seoul	No	1. Amox/clavulanic acid<br>2. Netilmicin	Clinical cure
Kangnam	P8	2020	Wound	Face	Yes	Yes	F	57	Seoul	No	1. Ampicillin sodium/sulbactam sodium<br>2. Sultamicillin	Clinical cure
Kangnam	P9	2021	Wound	Upper extremity	No	Yes	F	59	Seoul	No	1. Amox/clavulanic acid<br>2. Doxycycline<br>3. Ampicillin sodium/sulbactam sodium	Clinical cure
Kangnam	P10	2021	Wound	Face	Yes	Yes	M	48	Seoul	No	1. Ampicillin sodium/sulbactam sodium<br>2. Sultamicillin	Clinical cure
Kangnam	P11	2022	Wound	Face	No	Yes	F	32	Seoul	No	1. Ampicillin sodium/sulbactam sodium<br>2. Sultamicillin	Clinical cure
Kangnam	P12	2022	Wound	Face	No	Yes	F	52	Seoul	No	1. Cefalexin<br>2. Cefazolin<br>3. Ampicillin sodium/sulbactam sodium	Clinical cure

Hospital	Identification	Year	Specimen	Site	Polymicrobial infection	Animal contact	Sex	Age	Region	Hospitalization	Used antibiotics	Outcome
Kangnam	P13	2022	Wound	Upper extremity	No	Yes	M	49	Seoul	Yes	1. Ampicillin sodium/sulbactam sodium<br>2. Amox/clavulanic acid	Clinical cure
Hallym	P14	2018	Blood	Blood	No	No	M	59	Gyeonggi-do	Yes	Piperacillin/tazobactam	Clinical cure
Hallym	P15	2018	Wound	Lower extremity	No	Yes	M	55	Gyeonggi-do	No	Amox/clavulanic acid	Clinical cure
Hallym	P16	2018	Wound	Lower extremity	No	Yes	F	67	Gyeonggi-do	Yes	Amox/clavulanic acid	Clinical cure
Hallym	P17	2019	Wound	Upper extremity	No	Yes	M	30	Gyeonggi-do	Yes	Amox/clavulanic acid	Clinical cure
Hallym	P18	2019	Wound	Upper extremity	No	Yes	F	45	Gyeonggi-do	Yes	Amox/clavulanic acid	Clinical cure
Hallym	P19	2021	Wound	Upper extremity	No	Yes	F	68	Gyeonggi-do	Yes	Amox/clavulanic acid	Clinical cure
Ilsan	P20	2018	Wound	Upper extremity	No	Yes	F	71	Gyeonggi-do	No	Cefazolin	Clinical cure
Ilsan	P21	2018	Wound	Upper extremity	No	Yes	F	74	Gyeonggi-do	Yes	1. Cefazolin<br>2. Ampicillin-sulbactam<br>3. Sultamicillin	Clinical cure
sIlsan	P22	2021	Wound	Upper extremity	Yes	Yes	M	78	Gyeonggi-do	Yes	1. Cefazolin<br>2. Amikacin	Clinical cure
Ilsan	P23	2021	Wound	Upper extremity	No	Yes	F	54	Gyeonggi-do	No	1. Cefoxitin<br>2. Amoxacillin/clavulanic acid<br>3. Amikacin<br>4. Cefazolin<br>5. Piperacillin-tazobactam<br>6. Ampicillin sodium/sulbactam sodium	Clinical cure
Gseverance	P24	2019	Wound	Upper extremity	Yes	Yes	F	38	Gyeonggi-do	Yes	1. Ampicillin/sulbactam<br>2. Ceftriaxone	Clinical cure
Gseverance	P25	2019	Wound	Upper extremity	Yes	Yes	F	25	Seoul	No	1. Cefazolin<br>2. Amoxcillin/clavulanate	Clinical cure
Gseverance	P26	2019	Wound	Upper extremity	No	Yes	M	54	Seoul	No	1. Ceftriaxone<br>2. Cefdinir	Clinical cure
Gseverance	P27	2019	Wound	Upper extremity	No	Yes	M	54	Seoul	No	1. Ceftriaxone<br>2. Cefdinir	Clinical cure
Gseverance	P28	2019	Wound	Upper extremity	No	Yes	F	41	Seoul	Yes	1. Ampicillin/sulbactam<br>2. Levofloxacin<br>3. Sultamicillin	Clinical cure
Gseverance	P29	2019	Wound	Upper extremity	No	Yes	F	41	Seoul	Yes	1. Ampicillin/sulbactam<br>2. Levofloxacin<br>3. Sultamicillin	Clinical cure

Hospital	Identification	Year	Specimen	Site	Polymicrobial infection	Animal contact	Sex	Age	Region	Hospitalization	Used antibiotics	Outcome
Gseverance	P30	2019	Wound	Upper extremity	No	Yes	F	41	Seoul	Yes	1. Ampicillin/sulbactam 2. Levofloxacin 3. Sultamicillin	Clinical cure
Gseverance	P31	2019	Wound	Upper extremity	Yes	Yes	F	41	Seoul	Yes	1. Ampicillin/sulbactam 2. Levofloxacin 3. Sultamicillin	Clinical cure
Gseverance	P32	2019	Wound	Upper extremity	No	Yes	F	41	Seoul	Yes	1. Ampicillin/sulbactam 2. Levofloxacin 3. Sultamicillin	Clinical cure
Gseverance	P33	2019	Wound	Upper extremity	No	Yes	F	41	Seoul	Yes	1. Ampicillin/sulbactam 2. Levofloxacin 3. Sultamicillin	Clinical cure
Gseverance	P34	2019	Wound	Upper extremity	No	Yes	F	41	Seoul	Yes	1. Ampicillin/sulbactam 2. Levofloxacin 3. Sultamicillin	Clinical cure
Gseverance	P35	2021	Wound	Upper extremity	No	Yes	M	10	Seoul	No	1. Cefazolin 2. Cefadroxil	Clinical cure
Gseverance	P36	2022	Wound	Upper extremity	No	Yes	M	42	Seoul	Yes	1. Amikacin 2. Cefazolin 3. Amoxcillin/clavulanate	Clinical cure
Gseverance	P37	2022	Wound	Upper extremity	No	Yes	M	42	Seoul	Yes	1. Amikacin 2. Cefazolin 3. Amoxcillin/clavulanate	Clinical cure
Gseverance	P38	2022	Wound	Upper extremity	Yes	Yes	M	32	Seoul	Yes	1. Cefazolin 2. Amikacin 3. Ampicillin/sulbactam 4. Amoxcillin/clavulanate	Clinical cure
Gseverance	P39	2022	Wound	Upper extremity	Yes	Yes	M	32	Seoul	Yes	1. Cefazolin 2. Amikacin 3. Ampicillin/sulbactam 4. Amoxcillin/clavulanate	Clinical cure
Gseverance	P40	2022	Wound	Lower extremity	No	Yes	F	32	Seoul	No	Amoxcillin/clavulanate	Clinical cure
Gseverance	P41	2022	Wound	Upper extremity	No	Yes	F	40	Seoul	No	1. Clindamycin 2. Ampicillin/sulbactam 3. Amoxcillin/clavulanate	Clinical cure
Dongtan	P42	2018	Wound	Lower extremity	No	Yes	F	66	Seoul	No	1. Levofloxacin 2. Amikacin sulfate 3. Cefazedone Sodium	Clinical cure
Dongtan	P43	2018	Wound	Lower extremity	No	Yes	F	66	Gyeonggi-do	No	1. Levofloxacin 2. Amikacin sulfate 3. Cefazedone Sodium	Clinical cure
Dongtan	P44	2018	Wound	Lower extremity	No	Yes	F	54	Gyeonggi-do	Yes	1. Amoxicillin/dilute clavulanate potassium 2. Cefotaxime sodium 3. Levofloxacin	Clinical cure

Hospital	Identification	Year	Specimen	Site	Polymicrobial infection	Animal contact	Sex	Age	Region	Hospitalization	Used antibiotics	Outcome
Dongtan	P45	2018	Wound	Lower extremity	No	Yes	F	54	Gyeonggi-do	Yes	1. Ampicillin sodium/sulbactam sodium<br>2. Amox/clavulanic acid	Clinical cure
Dongtan	P46	2020	Wound	Lower extremity	No	Yes	F	49	Gyeonggi-do	Yes	1. Cefazolin sodium<br>2. Amoxicillin/dilute clavulanate potassium	Clinical cure
Dongtan	P47	2021	Wound	Upper extremity	No	Yes	M	34	Gyeonggi-do	Yes	1. Ceftriaxone sodium hydrate<br>2. Teicoplanin	Clinical cure
Dongtan	P48	2021	Wound	Upper extremity	No	Yes	M	34	Gyeonggi-do	Yes	1. Ceftriaxone sodium hydrate<br>2. Teicoplanin	Clinical cure
Sseverance	P49	2018	Blood	Blood	No	No	M	50	Jeollanam-do	Yes	1. Piperacillin/tazobactam<br>2. Moxifloxacin<br>3. Cefoperazone/sulbactam	Clinical cure
Sseverance	P50	2018	Wound	Upper extremity	Yes	Yes	M	66	Seoul	No	Amox/clavulanic acid	Clinical cure
Sseverance	P51	2018	Wound	Lower extremity	Yes	No	M	75	Seoul	Yes	Tazobactam	Clinical cure
Sseverance	P52	2018	Wound	Upper extremity	No	Yes	M	84	Seoul	No	None	Clinical cure
Sseverance	P53	2018	Wound	Upper extremity	No	Yes	F	87	Seoul	No	None	Clinical cure
Sseverance	P54	2018	Blood	Blood	Yes	No	F	62	Seoul	No	None	Clinical cure
Sseverance	P55	2019	Wound	Upper extremity	Yes	Yes	F	51	Seoul	No	1. Cephalosporins<br>2. Amox/clavulanic acid	Clinical cure
Sseverance	P56	2019	Wound	Upper extremity	Yes	Yes	F	36	Seoul	Yes	Amox/clavulanic acid	Clinical cure
Sseverance	P57	2020	Wound	Face	No	Yes	F	87	Seoul	No	1. Cefazolin<br>2. Amox/clavulanic acid	Clinical cure
Sseverance	P58	2020	CAPD fluid	CAPD fluid	No	No	M	71	Seoul	No	Cefazolin/sulbactam	Clinical cure
Sseverance	P59	2020	CAPD fluid	CAPD fluid	No	Yes	M	71	Seoul	No	1. Ceftriaxone/sulbactam<br>2. Vancomycin<br>3. Amikacin<br>4. Doxycycline<br>5. Rifampin<br>6. Fluconazole<br>7. Moxifloxacin	Clinical cure
Sseverance	P60	2021	Wound	Upper extremity	No	Yes	F	92	Seoul	No	1. Amoxaciliin<br>2. Cefdinir	Clinical cure
Sseverance	P61	2021	Blood	Blood	No	No	F	84	Seoul	Yes	1. Piperacillin/tazobactam<br>2. Levofloxacin	Death

Hospital	Identification	Year	Specimen	Site	Polymicrobial infection	Animal contact	Sex	Age	Region	Hospitalization	Used antibiotics	Outcome
											3. Meropenem<br>4. Teicoplanin	
Sseverance	P62	2021	Wound	Upper extremity	Yes	Yes	F	52	Seoul	No	1. Tazobactam<br>2. Sulbactam<br>3. Cephalosporins<br>4. Amox/clavulanic acid	Clinical cure
Sseverance	P63	2021	Wound	Upper extremity	Yes	Yes	F	84	Seoul	No	1. Sulbactam<br>2. Amox/clavulanic acid	Clinical cure
Gangdong	P65	2022	Wound	Lower extremity	No	Yes	F	46	Gyeonggi-do	Yes	Cephalosporins	Clinical cure
Gangdong	P66	2022	Wound	Lower extremity	Yes	No	M	72	Jeollabuk-do	Yes	Ceftriaxone	Clinical cure
Gangdong	P67	2022	Wound	Lower extremity	No	Yes	M	55	Seoul	Yes	Amox/clavulanic acid	Clinical cure
Gangdong	P68	2021	Wound	Upper extremity	No	Yes	M	33	Seoul	No	Amox/clavulanic acid	Clinical cure
Gangdong	P70	2021	Wound	Upper extremity	No	Yes	F	49	Seoul	Yes	Amox/clavulanic acid	Clinical cure
Gangdong	P71	2021	Wound	Upper extremity	No	Yes	F	37	Seoul	Yes	1. Amox/clavulanic acid<br>2. Moxifloxacin	Clinical cure
Gangdong	P72	2021	Wound	Upper extremity	No	Yes	F	52	Gyeonggi-do	Yes	Amox/clavulanic acid	Clinical cure
SCL	P73	2018	Pus				M	74	Jeollanam-do			
SCL	P74	2018	Pus				F	77	Chungcheongbuk-do			
SCL	P75	2018	Wound				M	47	Gyeongsangbuk-do			
SCL	P76	2018	Pus				M	55	Gyeongsangnam-do			
SCL	P77	2018	Pus				F	30	Gyeongsangbuk-do			
SCL	P78	2018	Pus				M	34	Gyeongsangnam-do			
SCL	P79	2018	Wound				F	41	Gyeongsangnam-do			
SCL	P80	2018	Pus				M	30	Gyeongsangbuk-do			
SCL	P82	2018	Wound				M	65	Gyeongsangnam-do			
SCL	P84	2018	Pus				M	37	Chungcheongnam-do			
SCL	P86	2018	Wound				F	94	Gyeongsangbuk-do			
SCL	P87	2018	Wound				M	22	Gyeongsangnam-do			
SCL	P88	2018	Wound				F	50	Gyeonggi-do			
SCL	P89	2018	Wound				M	46	Chungcheongbuk-do			
SCL	P91	2018	Pus				M	43	Gyeongsangnam-do			
SCL	P92	2018	Pus				F	51	Gyeongsangbuk-do			
SCL	P93	2018	Wound				F	59	Gyeongsangbuk-do			
SCL	P94	2018	Wound				M	58	Seoul			
SCL	P96	2018	Wound				F	62	Gyeongsangbuk-do			
SCL	P97	2018	Wound				F	46	Gyeongsangnam-do			
SCL	P98	2018	Pus				M	36	Jeollanam-do			
SCL	P100	2018	Wound				F	49	Gyeongsangnam-do			
SCL	P101	2018	Wound				F	43	Chungcheongbuk-do			
SCL	P102	2018	Pus				F	23	Seoul			
SCL	P103	2018	Wound				F	26	Jeollanam-do			
SCL	P104	2018	Fluid				F	57	Gyeongsangbuk-do			

Hospital	Identification	Year	Specimen	Site	Polymicrobial infection	Animal contact	Sex	Age	Region	Hospitalization	Used antibiotics	Outcome
SCL	P105	2018	Fluid				M	64	Gyeongsangnam-do			
SCL	P106	2018	Wound				F	47	Gyeongsangbuk-do			
SCL	P107	2018	Pus				F	77	Seoul			
SCL	P108	2018	Wound				F	62	Gyeongsangbuk-do			
SCL	P110	2019	Pus				F	39	Gyeongsangnam-do			
SCL	P111	2019	Wound				F	62	Gyeongsangbuk-do			
SCL	P113	2019	Wound				M	75	Seoul			
SCL	P114	2019	Wound				F	65	Gyeongsangbuk-do			
SCL	P115	2019	Pus				M	61	Jeollanam-do			
SCL	P116	2019	Wound				F	38	Gyeongsangbuk-do			
SCL	P117	2019	Wound				M	38	Chungcheongbuk-do			
SCL	P118	2019	Pus				F	54	Gyeongsangbuk-do			
SCL	P119	2019	Wound				F	30	Gyeongsangnam-do			
SCL	P120	2019	Pus				M	66	Gyeongsangnam-do			
SCL	P121	2019	Blood				M	48	Incheon			
SCL	P123	2019	Wound				F	27	Gyeonggi-do			
SCL	P124	2019	Wound				F	39	Incheon			
SCL	P125	2019	Pus				F	41	Seoul			
SCL	P126	2019	Pus				M	77	Gyeongsangbuk-do			
SCL	P127	2019	Wound				F	72	Gyeongsangnam-do			
SCL	P128	2019	Blood				F	89	Jeollabuk-do			
SCL	P129	2019	Pus				F	59	Gyeongsangnam-do			
SCL	P130	2019	Pus				M	8	Gyeongsangbuk-do			
SCL	P131	2019	Wound				M	21	Gyeongsangbuk-do			
SCL	P132	2019	Pus				F	81	Gyeongsangnam-do			
SCL	P133	2019	Wound				F	84	Gyeongsangbuk-do			
SCL	P134	2019	Wound				M	45	Chungcheongbuk-do			
SCL	P135	2019	Wound				F	74	Gyeongsangnam-do			
SCL	P136	2019	Wound				M	45	Gyeonggi-do			
SCL	P137	2019	Wound				M	10	Chungcheongbuk-do			
SCL	P138	2019	Pus				F	61	Jeollanam-do			
SCL	P139	2019	Wound				M	68	Chungcheongbuk-do			
SCL	P140	2019	Pus				F	53	Gyeongsangnam-do			
SCL	P141	2019	Pus				F	50	Gyeongsangbuk-do			
SCL	P142	2019	Wound				F	25	Gyeongsangnam-do			
SCL	P143	2019	Wound				F	28	Chungcheongbuk-do			
SCL	P144	2019	Pus				F	79	Gyeonggi-do			
SCL	P145	2019	Pus				F	45	Gyeongsangnam-do			
SCL	P146	2019	Pus				M	57	Jeollanam-do			
SCL	P147	2019	Pus				F	62	Gyeongsangbuk-do			
SCL	P148	2019	Pus				M	58	Gyeongsangnam-do			
SCL	P150	2019	Wound				F	65	Seoul			
SCL	P151	2019	Wound				M	40	Gyeongsangnam-do			
SCL	P153	2020	Pus				F	53	Seoul			
SCL	P154	2020	Wound				F	82	Gyeongsangbuk-do			
SCL	P155	2020	Wound				F	60	Chungcheongnam-do			
SCL	P156	2020	Wound				M	82	Gyeongsangnam-do			
SCL	P157	2020	Wound				M	46	Gyeongsangbuk-do			

Hospital	Identification	Year	Specimen	Site	Polymicrobial infection	Animal contact	Sex	Age	Region	Hospitalization	Used antibiotics	Outcome
SCL	P158	2020	Wound				F	50	Gyeongsangbuk-do			
SCL	P159	2020	Pus				F	52	Gyeongsangnam-do			
SCL	P160	2020	Pus				F	40	Jeollanam-do			
SCL	P161	2020	Wound				F	60	Seoul			
SCL	P162	2020	Pus				F	58	Gyeonggi-do			
SCL	P163	2020	Pus				F	40	Gyeongsangbuk-do			
SCL	P164	2020	Wound				F	31	Gyeonggi-do			
SCL	P165	2020	Pus				F	31	Jeollanam-do			
SCL	P166	2020	Wound				F	29	Gyeongsangbuk-do			
SCL	P168	2020	Wound				M	50	Gyeongsangbuk-do			
SCL	P169	2020	Wound				M	49	Gyeongsangnam-do			
SCL	P170	2020	Wound				F	22	Gyeonggi-do			
SCL	P171	2020	Wound				F	48	Gyeonggi-do			
SCL	P172	2020	Wound				F	25	Gyeongsangbuk-do			
SCL	P173	2020	Pus				F	62	Jeollanam-do			
SCL	P174	2020	Pus				F	42	Chungcheongbuk-do			
SCL	P175	2020	Fluid				F	86	Gyeongsangnam-do			
SCL	P176	2020	Wound				M	58	Chungcheongbuk-do			
SCL	P177	2020	Pus				F	79	Gyeongsangnam-do			
SCL	P178	2020	Wound				F	84	Chungcheongbuk-do			
SCL	P180	2020	Pus				F	59	Gyeongsangbuk-do			
SCL	P181	2020	Pus				F	78	Gyeongsangnam-do			
SCL	P182	2020	Pus				F	84	Chungcheongbuk-do			
SCL	P184	2020	Pus				F	74	Gyeongsangnam-do			
SCL	P185	2020	Wound				F	38	Gyeongsangbuk-do			
SCL	P186	2020	Pus				M	53	Chungcheongbuk-do			
SCL	P187	2020	Wound				M	77	Gyeongsangbuk-do			
SCL	P188	2020	Wound				M	61	Chungcheongbuk-do			
SCL	P189	2020	Pus				M	57	Gyeongsangbuk-do			
SCL	P190	2020	Wound				F	61	Jeollabuk-do			
SCL	P191	2020	Blood				F	80	Seoul			
SCL	P192	2020	Pus				M	43	Gyeonggi-do			
SCL	P193	2020	Wound				M	59	Gyeongsangbuk-do			
SCL	P194	2021	Pus				M	16	Gyeongsangnam-do			
SCL	P195	2021	Wound				F	73	Gyeongsangnam-do			
SCL	P196	2021	Pus				F	55	Jeollanam-do			
SCL	P197	2021	Pus				M	32	Gyeonggi-do			
SCL	P198	2021	Wound				F	82	Gyeongsangnam-do			
SCL	P199	2021	Wound				M	60	Jeollanam-do			
SCL	P200	2021	Other				M	72	Gyeonggi-do			
SCL	P201	2021	Other				M	35	Seoul			
SCL	P204	2021	Wound				F	52	Chungcheongbuk-do			
SCL	P205	2021	Wound				F	77	Gyeongsangnam-do			
SCL	P206	2021	Pus				M	58	Gyeongsangbuk-do			
SCL	P207	2021	Pus				F	45	Seoul			
SCL	P208	2021	Pus				F	30	Gyeongsangnam-do			
SCL	P209	2021	Wound				F	51	Gyeongsangbuk-do			
SCL	P211	2021	Pus				F	86	Chungcheongbuk-do			

Hospital	Identification	Year	Specimen	Site	Polymicrobial infection	Animal contact	Sex	Age	Region	Hospitalization	Used antibiotics	Outcome
SCL	P212	2021	Wound				M	39	Gyeongsangbuk-do			
SCL	P214	2021	Wound				F	51	Gyeongsangnam-do			
SCL	P215	2021	Other				M	67	Chungcheongbuk-do			
SCL	P216	2021	Pus				M	21	Jeollanam-do			
SCL	P217	2021	Wound				F	30	Incheon			
SCL	P218	2021	Pus				M	24	Gyeongsangnam-do			
SCL	P221	2021	Wound				F	71	Gyeongsangbuk-do			
SCL	P224	2021	Wound				F	27	Gyeongsangbuk-do			
SCL	P225	2021	Pus				F	46	Seoul			
SCL	P226	2021	Wound				F	27	Chungcheongbuk-do			
SCL	P227	2021	Wound				M	38	Gyeonggi-do			
SCL	P229	2021	Wound				F	52	Incheon			
SCL	P230	2021	Wound				F	54	Gyeongsangnam-do			
SCL	P231	2021	Pus				F	51	Gyeongsangbuk-do			
SCL	P232	2021	Pus				F	54	Gyeonggi-do			
SCL	P233	2021	Pus				F	41	Gyeonggi-do			
SCL	P234	2021	Pus				F	71	Jeollanam-do			
SCL	P235	2021	Wound				F	55	Gyeongsangbuk-do			
SCL	P236	2021	Wound				F	52	Chungcheongbuk-do			
SCL	P237	2021	Wound				F	55	Chungcheongbuk-do			
SCL	P238	2021	Pus				M	49	Gyeongsangnam-do			
SCL	P239	2021	Pus				F	24	Gyeongsangnam-do			
SCL	P240	2021	Pus				M	55	Gyeongsangnam-do			
SCL	P241	2021	Wound				F	89	Jeollanam-do			
SCL	P242	2021	Pus				F	60	Gyeongsangnam-do			
SCL	P243	2021	Wound				F	82	Gyeongsangbuk-do			
SCL	P244	2021	Pus				F	38	Jeollanam-do			
SCL	P246	2021	Other				F	67	Chungcheongbuk-do			
SCL	P247	2021	Pus				F	82	Gyeongsangnam-do			
SCL	P248	2021	Wound				F	55	Chungcheongbuk-do			
SCL	P249	2021	Pus				F	19	Seoul			
SCL	P250	2022	Wound				F	41	Gyeongsangbuk-do			
SCL	P251	2022	Wound				M	51	Gyeongsangnam-do			
SCL	P252	2022	Pus				M	63	Gyeongsangnam-do			
SCL	P253	2022	Wound				M	54	Gyeonggi-do			
SCL	P254	2022	Wound				F	75	Gyeongsangnam-do			
SCL	P255	2022	Wound				M	79	Gyeongsangbuk-do			
SCL	P256	2022	Wound				F	86	Gyeonggi-do			
SCL	P257	2022	Wound				F	79	Gyeongsangbuk-do			
SCL	P258	2022	Wound				M	73	Jeollanam-do			
SCL	P259	2022	Pus				M	63	Gyeongsangbuk-do			
SCL	P260	2022	Pus				F	72	Jeju			
SCL	P261	2022	Wound				F	46	Gyeongsangbuk-do			
SCL	P263	2022	Pus				F	43	Seoul			
SCL	P264	2022	Pus				F	44	Gyeongsangbuk-do			
SCL	P265	2022	Pus				F	48	Gyeongsangnam-do			
SCL	P266	2022	Pus				F	30	Gyeongsangnam-do			
SCL	P269	2022	Pus				F	34	Seoul			

Hospital	Identification	Year	Specimen	Site	Polymicrobial infection	Animal contact	Sex	Age	Region	Hospitalization	Used antibiotics	Outcome
SCL	P270	2022	Pus				F	46	Gyeongsangnam-do			
SCL	P271	2022	Pus				F	68	Jeollanam-do			
SCL	P272	2022	Pus				F	43	Gyeongsangbuk-do			
SCL	P273	2022	Other				F	43	Gyeongsangbuk-do			
SCL	P274	2022	Wound				F	65	Gyeongsangbuk-do			
SCL	P275	2022	Pus				F	50	Gyeongsangnam-do			
SCL	P276	2022	Wound				F	93	Gyeongsangnam-do			
SCL	P277	2022	Wound				M	36	Chungcheongbuk-do			
SCL	P278	2022	Pus				F	60	Gyeongsangbuk-do			
SCL	P281	2022	Wound				F	82	Incheon			
SCL	P282	2022	Pus				F	73	Incheon			
SCL	P283	2022	Wound				M	49	Jeollanam-do			
SCL	P284	2022	Wound				F	37	Gyeongsangbuk-do			
SCL	P285	2022	Wound				M	48	Gyeongsangnam-do			
SCL	P286	2022	Wound				M	66	Chungcheongbuk-do			
SCL	P287	2022	Wound				F	65	Gyeongsangnam-do			
SCL	P288	2022	Wound				F	85	Gyeongsangnam-do			
SCL	P289	2022	Wound				F	19	Gyeongsangbuk-do			
SCL	P290	2022	Pus				F	70	Seoul			
SCL	P292	2022	Pus				F	27	Gyeonggi-do			
SCL	P293	2022	Wound				F	50	Gyeongsangnam-do			
SCL	P294	2022	Wound				F	55	Chungcheongbuk-do			
SCL	P295	2022	Wound				M	61	Chungcheongbuk-do			
SCL	P296	2022	Wound				M	76	Chungcheongbuk-do			
SCL	P297	2022	Pus				F	13	Jeollabuk-do			
SCL	P298	2022	Wound				F	90	Gyeongsangbuk-do			
SCL	P299	2022	Pus				F	23	Gyeongsangnam-do			
SCL	P300	2022	Wound				F	62	Incheon			
SCL	P301	2022	Wound				F	38	Chungcheongbuk-do			
SCL	P302	2022	Wound				F	38	Seoul			
SCL	P303	2022	Pus				F	40	Gyeongsangnam-do			
SCL	P304	2022	Wound				M	85	Chungcheongbuk-do			
SCL	P305	2022	Wound				M	53	Jeollanam-do			
SCL	P307	2022	Wound				F	64	Jeollanam-do			
SCL	P308	2022	Wound				M	69	Gyeongsangbuk-do			
SCL	P309	2022	Pus				F	45	Gyeonggi-do			
SCL	P310	2022	Wound				F	26	Gyeongsangbuk-do			
SCL	P311	2022	Pus				M	50	Jeollanam-do			
SCL	P312	2022	Wound				F	54	Incheon			
SCL	P313	2022	Pus				M	22	Gyeongsangnam-do			
SCL	P314	2022	Pus				F	45	Gyeongsangnam-do			
SCL	P315	2022	Wound				F	51	Jeollanam-do			
SCL	P316	2022	Pus				F	29	Seoul			

Appendix Table 5. Dataset of the global prevalence of infections caused by *Pasteurella* species for meta-analysis

Study ID	Detected total	Blood positive	Published year	Location
Athanasia et al. [2005]	13	3	Before 2010	Greece
Dernoncourt et al. [2022]	215	14	After 2010	Europe
Ebright et al. [2009]	179	14	Before 2010	USA
Escande et al. [1993]	958	102	Before 2010	Europe
Giordano et al. [2015]	44	8	After 2010	USA
Holst et al. [1992]	146	5	Before 2010	Denmark
Kormondi et al. [2018]	162	14	After 2010	Europe
Mahony et al. [2023]	190	22	After 2010	Australia
Nollet et al. [2016]	28	8	After 2010	Europe
Nseir et al. [2009]	77	25	Before 2010	Israel

Appendix Table 6. Characteristics of patients with *Pasteurella* species infection

Characteristics	Value*
Age	52.0 (40.0-66.0)
Sex	
Male	95 (33.6%)
Female	188 (66.4%)
Animal contact	
Yes	62 (88.6%)
No	8 (11.4%)
Polymicrobial	
Yes	18 (25.7%)
No	52 (74.3%)
Year	
2018	46 (16.3%)
2019	55 (19.4%)
2020	48 (17.0%)
2021	62 (21.9%)
2022	72 (25.4%)
Hospitalization	
In-patient	38 (54.3%)
Out-patient	32 (45.7%)

*Values are expressed as the median (1st to 3rd quartile range) or no. (%).

Appendix Table 7. Comparison of the characteristics of patients with *Pasteurella* species infections who were or were not hospitalized*

Characteristics	In-patient	Out-patient	Total	*P*-value
Age	47.5 (41.0–59.0)	54.0 (34.0–68.5)	51.0 (40.0–66.0)	0.443
Sex				0.848
Male	15 (39.5%)	11 (34.4%)	26 (37.1%)	
Female	23 (60.5%)	21 (65.6%)	44 (62.9%)	
Animal contact				0.383
Yes	32 (84.2%)	30 (93.8%)	62 (88.6%)	
No	6 (15.8%)	2 (6.2%)	8 (11.4%)	
Specimen				0.156
Blood	4 (10.5%)	1 (3.1%)	5 (7.1%)	
CAPD fluid	0 (0.0%)	2 (6.2%)	2 (2.9%)	
Wound	34 (89.5%)	29 (90.6%)	63 (90.0%)	
Polymicrobial				0.485
Yes	8 (21.1%)	10 (31.2%)	18 (25.7%)	
No	30 (78.9%)	22 (68.8%)	52 (74.3%)	
Year				0.152
2018	7 (18.4%)	9 (28.1%)	16 (22.9%)	
2019	12 (31.6%)	4 (12.5%)	16 (22.9%)	
2020	3 (7.9%)	7 (21.9%)	10 (14.3%)	
2021	8 (21.1%)	8 (25.0%)	16 (22.9%)	
2022	8 (21.1%)	4 (12.5%)	12 (17.1%)	
Bacteremia				0.464
Yes	4 (10.5%)	1 (3.1%)	5 (7.1%)	
No	34 (89.5%)	31 (96.9%)	65 (92.9%)	

*Values are expressed as median (1st to 3rd quartile range) or no. (%).

Appendix Table 8. Comparison of the characteristics of patients with *Pasteurella* infection with or without bacteremia*

Characteristics	Bacteremia	Non-bacteremia	*P*-value
Age, years	68.5 (54.5-82.0)	52.0 (39.5-65.5)	0.021
Sex			1.000
Male	3 (37.5%)	92 (33.5%)	

Characteristics	Bacteremia	Non-bacteremia	*P*-value
Female	5 (62.5%)	183 (66.5%)	
Animal contact			<0.001
Yes	0 (0.0%)	62 (95.4%)	
No	5 (100.0%)	3 (4.6%)	
Polymicrobial			1.000
Yes	1 (20.0%)	17 (26.2%)	
No	4 (80.0%)	48 (73.8%)	
Year			0.271
2018	3 (37.5%)	43 (15.6%)	
2019	2 (25.0%)	53 (19.3%)	
2020	2 (25.0%)	46 (16.7%)	
2021	1 (12.5%)	61 (22.2%)	
2022	0 (0.0%)	72 (26.2%)	
Hospitalization			0.464
In-patient	4 (80.0%)	34 (52.3%)	
Out-patient	1 (20.0%)	31 (47.7%)	

*****Values are expressed as the median (1st to 3rd quartile range) or number (%). For age, sex, and year, the number of bacteraemia and non-bacteraemia cases were 8 and 275 from all cases of hospitals and the reference laboratory, respectively.

Appendix Table 9. Sensitivity analyses of the included data for the prevalence of bacteremia caused by *Pasteurella* species

Omitted study	Estimated prevalence (%)	95% confidence interval (%)
Athanasia et al. [2005]	12.1	6.9–18.4
Dernoncourt et al. [2022]	13.4	7.6–20.4
Ebright et al. [2009]	13.2	7.3–20.3
Escande et al. [1993]	12.9	6.9–20.2
Giordano et al. [2015]	12.0	6.5–18.7
Holst et al. [1992]	13.8	8.4–20.2
Kormondi et al. [2018]	13.1	7.2–20.3
Mahony et al. [2023]	12.7	6.8–19.9
Nollet et al. [2016]	11.3	6.5–17.1
Nseir et al. [2009]	9.9	6.4–13.9

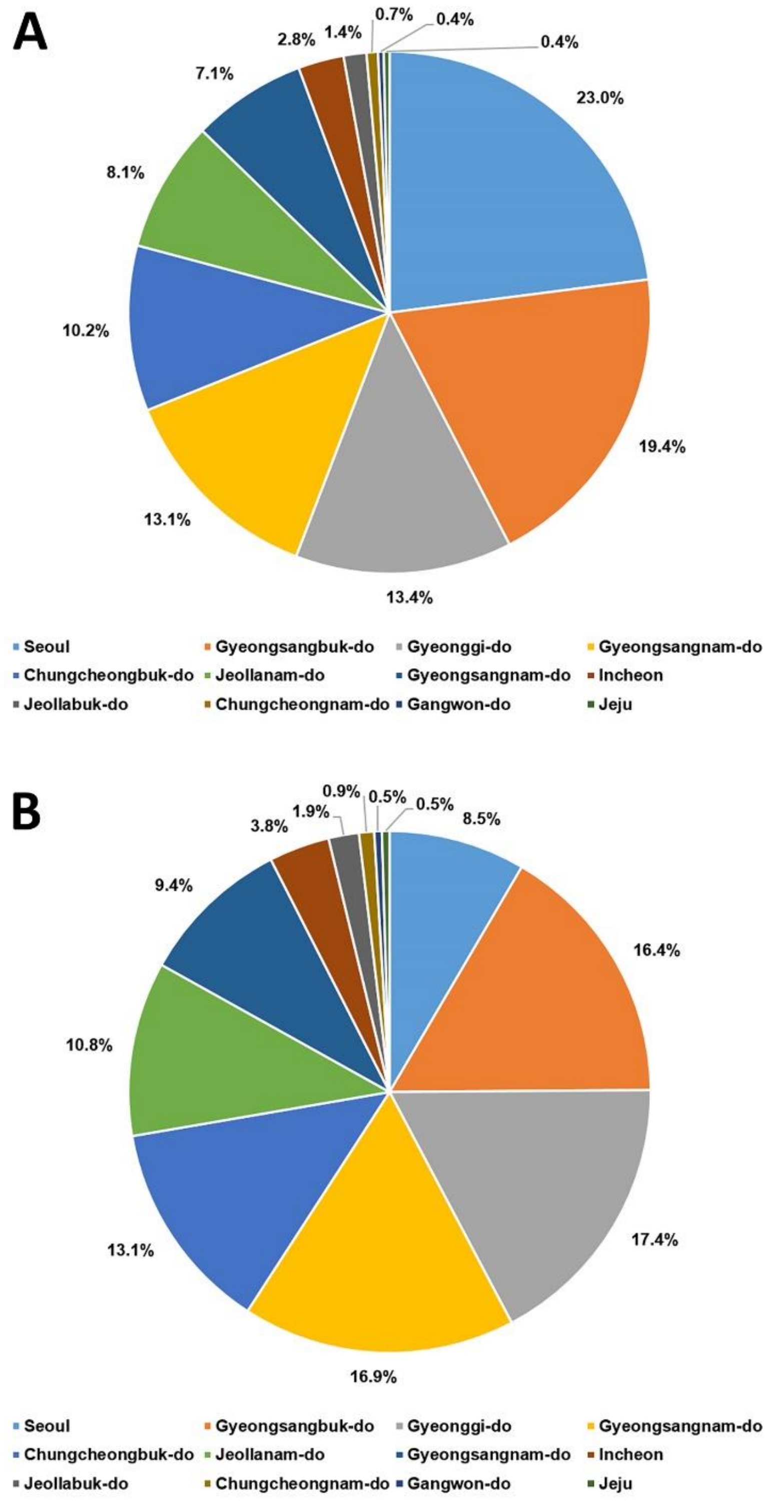

Appendix Figure 1. Distribution of *Pasteurella* infections stratified by regions. A) All 283 cases from hospitals and a reference laboratory. B) Only 213 cases from a reference laboratory.

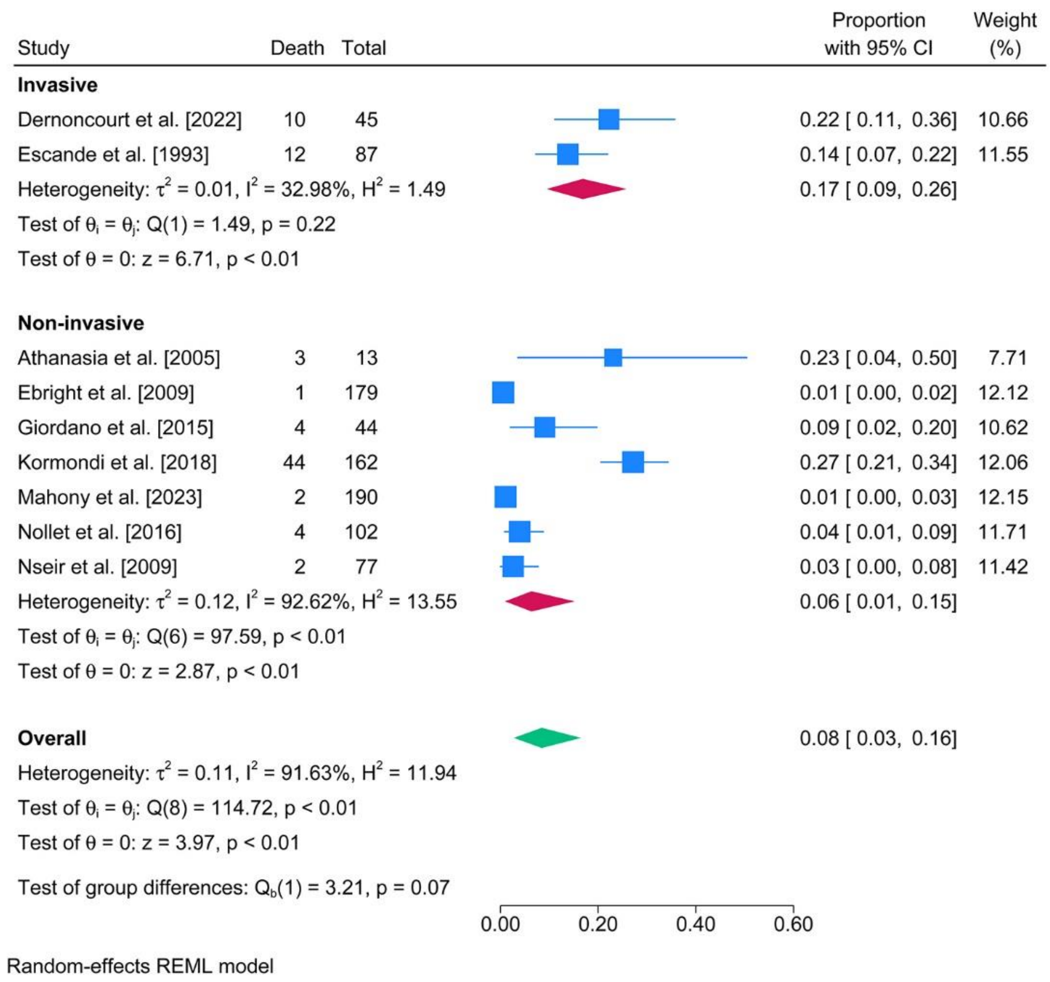

Appendix Figure 2. Forest plots for the pooled death rates in patients with *Pasteurella* infections.

References

1. Chandranaik BM, Hegde R, Shivashankar BP, Giridhar P, Muniyellappa HK, Kalge R, et al. Serotyping of foot and mouth disease virus and Pasteurella multocida from Indian gaurs (Bos gaurus), concurrently infected with foot and mouth disease and haemorrhagic septicaemia. Trop Anim Health Prod. 2015;47:933–7. https://doi.org/10.1007/s11250-015-0811-x

2. Chomnawang MT, Nabnuengsap J, Kittiworakarn J, Pathanasophon P. Expression and immunoprotective property of a 39-kDa PlpB protein of Pasteurella multocida. J Vet Med Sci. 2009;71:1479–85. https://doi.org/10.1292/jvms.001779

3. Dunbar MR, Wolcott MJ, Rimler RB, Berlowski BM. Septicemic pasteurellosis in free-ranging neonatal pronghorn in Oregon. J Wildl Dis. 2000;36:383–8. https://doi.org/10.7589/0090-3558-36.2.383

4. Kawasaki M, Young JR, Suon S, Bush RD, Windsor PA. The Socioeconomic Impacts of Clinically Diagnosed Haemorrhagic Septicaemia on Smallholder Large Ruminant Farmers in Cambodia. Transbound Emerg Dis. 2015;62:535–48. https://doi.org/10.1111/tbed.12174

5. Martrenchar A, Njanpop BM. Première observation d'une épidémie de septicémie hémorragique due à Pasteurella multocida sérotype B6 au Nord-Cameroun. Rev Élev Méd Vét Pays Trop. 1994;47:19–20. https://doi.org/10.19182/remvt.9126

6. Moustafa AM, Bennett MD, Edwards J, Azim K, Mesaik MA, Choudhary MI, et al. Molecular typing of haemorrhagic septicaemia-associated Pasteurella multocida isolates from Pakistan and Thailand using multilocus sequence typing and pulsed-field gel electrophoresis. Res Vet Sci. 2013;95:986–90. https://doi.org/10.1016/j.rvsc.2013.07.003

7. Qudratullah MG, Saqib M, Bilal MQ. Isolation, characterization, virulence and immunogenicity testing of field isolates of Pasteurella multocida, Staphylococcus aureus, and Streptococcus agalactiae in laboratory settings. Acta Trop. 2017;172:70–4. https://doi.org/10.1016/j.actatropica.2017.04.020

8. Sarangi LN, Thomas P, Gupta SK, Kumar S, Viswas KN, Singh VP. Molecular Epidemiology of Pasteurella multocida Circulating in India by Multilocus Sequence Typing. Transbound Emerg Dis. 2016;63:e286–92. https://doi.org/10.1111/tbed.12270

9. Voigts A, Ngaisiue G, Henton MM, Hübschle OJ. Haemorrhagic septicaemia due to Pasteurella multocida type B2 in Namibia. Trop Anim Health Prod. 1997;29:247–8. https://doi.org/10.1007/BF02632315

10. Prakash V, Yun H. Pasteurella multocida epiglottitis and bacteremia in a patient with chronic lymphocytic leukemia: Case report and review of the literature. Infect Dis Clin Pract. 2009;17:124–6. https://doi.org/10.1097/IPC.0b013e31817cfd8d

11. Levy CE, Irino K, Funayama CR, Moura-Ribeiro MV. Meningencefalite por Pasteurella multocida: estudo clínico-laboratorial de um caso em lactente. Arq Neuropsiquiatr. 1989;47:468–70. https://doi.org/10.1590/S0004-282X1989000400014

12. Bardhan D, Kumar S, Sekaran GA, Meraj M, Chilambarasan M, Singh RK, et al. Economic losses due to hemorrhagic septicaemia in India. Indian J Anim Sci. 2020;90:18–23. https://doi.org/10.56093/ijans.v90i3.102320

13. Biswas A, Shivachandra SB, Saxena MK, Kumar AA, Singh VP, Srivastava SK. Molecular variability among strains of Pasteurella multocida isolated from an outbreak of haemorrhagic septicaemia in

India. Vet Res Commun. 2004;28:287–98. https://doi.org/10.1023/B:VERC.0000026656.77847.5b

14. MacPhillamy I, Young J, Siek S, Bun C, Suon S, Toribio JA, et al. Improving Village Animal Health Worker participation in national disease surveillance systems: A case study from Cambodia. Transbound Emerg Dis. 2020;67:967–78. https://doi.org/10.1111/tbed.13432

15. Mondal SP, Yamage M. A retrospective study on the epidemiology of anthrax, foot and mouth disease, haemorrhagic septicaemia, peste des petits ruminants and rabies in Bangladesh, 2010–2012. PLoS One. 2014;9:e104435. https://doi.org/10.1371/journal.pone.0104435

16. Tomer P, Chaturvedi GC, Minakshi, Malik, Monga. Minakshi, Malik P, Monga DP. Comparative analysis of the outer membrane protein profiles of isolates of the Pasteurella multocida (B:2) associated with haemorrhagic septicaemia. Vet Res Commun. 2002;26:513–22. https://doi.org/10.1023/A:1020212430041

17. Kannangara DW, Pandya D, Patel P. Pasteurella multocida Infections with Unusual Modes of Transmission from Animals to Humans: A Study of 79 Cases with 34 Nonbite Transmissions. Vector Borne Zoonotic Dis. 2020;20:637–51. https://doi.org/10.1089/vbz.2019.2558

18. Bhonsle MG. Unusual incidence of haemorrhagic septicaemia. Indian Vet J. 1951;27:285–6.

19. Carter GR. Whatever happened to hemorrhagic septicemia? J Am Vet Med Assoc. 1982;180:1176–7.

20. Martrenchar A. Haemorrhagic septicaemia in Cameroon. Vet Rec. 1993;133:628.

21. Rimler RB, Wilson MA. Re-examination of Pasteurella multocida serotypes that caused haemorrhagic septicaemia in North America. Vet Rec. 1994;134:256. https://doi.org/10.1136/vr.134.10.256
